# Garlic exerts allelopathic effects on pepper physiology in a hydroponic co-culture system

**DOI:** 10.1242/bio.016451

**Published:** 2016-04-19

**Authors:** Haiyan Ding, Zhihui Cheng, Menglong Liu, Sikandar Hayat, Han Feng

**Affiliations:** College of Horticulture, Northwest A&F University, Yangling, Shannxi 712100, China

**Keywords:** Garlic, Pepper, Co-culture, Root exudates, Allelopathy

## Abstract

A hydroponic co-culture system was adopted to determine the allelopathic potential of garlic on the growth of pepper plants. Different numbers of garlic plants (0, 2, 4, 8 and 12) were hydroponically co-cultured with two pepper plants to investigate allelopathic effects on the growth attributes and antioxidative defense system of the test pepper plants. The responses of the pepper plants depended on the number of garlic plants included in the co-culture system, indicating an association of pepper growth with the garlic root exudate concentration. When grown at a pepper/garlic ratio of 1:1 or 1:2, the pepper plant height, chlorophyll content, and peroxidase (POD), catalase (CAT) and phenylalanine ammonia-lyase (PAL) activities were significantly increased after 30 days of co-culture; in contrast, reduction in methane dicarboxylic aldehyde (MDA) content was observed. However, when the pepper/garlic ratio was 1:4 or higher, these morphological indices and protective enzyme activities were significantly inhibited, whereas MDA levels in the pepper leaves were significantly increased due to severe membrane lipid peroxidation. The results indicate that although low concentrations of garlic root exudates appear to induce protective enzyme systems and promote pepper growth, high concentrations have deleterious effects. These findings suggest that further investigations should optimize the co-culture pepper/garlic ratio to reduce continuous cropping obstacles in pepper production.

## Abstract (precis)

**Summary:** Garlic exerts allelopathic effects on pepper physiology in a hydroponic co-culture system, with low concentrations of garlic root promoting pepper growth, but high concentrations having deleterious effects.

## INTRODUCTION

Allelopathy is defined as the positive or negative effects of one plant (as well as microorganisms) on the growth of another plant via the active release of chemical compounds into the environment through root exudation, leaching, volatilization as well as passive release through decomposition. These chemical compounds are defined as allelochemicals (Oracz et al., 2007; Inderjit and Duke, 2003) and include secondary metabolites and phytochemicals belonging to the following classes; flavonoid, terpenoid, phenolic compound, organic cyanide, glucosinolate, saponin, alkaloid and long chain fatty acid structures. Allelochemicals differ among species, organs and tissues (Ambika, 2013; Gniazdowska and Bogatek, 2005). The structure and concentration of allelochemicals varies with the biological and non-biological cues, therefore, their targets and functions are different (Bais, 2003).

Similar to other bioactive factors, allelochemicals target specific physiological processes. The modes of their action include disrupting membrane permeability, iron uptake, inhibiting photosynthetic and respiratory chain electron transport, cell division and ultrastructure, altering enzyme activity, and altering the balance between antioxidant defences and reactive oxygen species (ROS) levels (Gniazdowska and Bogatek, 2005). ROS in plants are highly reactive and toxic, causing potential damage to proteins, lipids, carbohydrates and DNA resulting in oxidative stress (Radhika et al. 2005). To protect cells from oxidative damage, plants possess highly efficient enzymatic antioxidant defence systems for scavenging ROS (Gill and Tuteja, 2010; Talukdar, 2013). Superoxide dismutase (SOD) is a ubiquitous organic molecule which is involved in regulation of oxygen metabolism in plants. As the first line of defence, SOD prevents ROS damage, controls lipid peroxidation and reduces damage to the membrane system by scavenging O_2_^−^ and converting it to H_2_O_2_ and O_2_ (Smirnoff, 2005). H_2_O_2_, a strong oxidant that can produce highly active OH^−^ via the Haber–Weiss reaction, can be decomposed into H_2_O and O_2_ by peroxidase (POD) and catalase (CAT), thereby preventing potential ROS damage to plants (Rakhra et al., 2015). In addition, the activity of phenylalanine ammonia-lyase (PAL), which plays an important role in secondary metabolism in plants, greatly increases upon infection and stress (Godinez-Vidal et al., 2008; Wada et al., 2014). Methane dicarboxylic aldehyde (MDA) is a decomposition product of the polyunsaturated fatty acid hydroperoxides that are generated from reactions with ROS (Tommasino et al., 2012). As it is a reactive aldehyde, the extent of membrane lipid peroxidation can be detected by measuring the content of MDA in an organism (Davey et al., 2005). Hence, the MDA level is considered an indirect index of the extent of damage in plants or their resistance to such damage (Gao, 2006).

In allelopathy research, different bioassay techniques such as the tube method, modified sponge-dish method, filter paper method, plant root box method, field method, pot culture method, agar culture method etc., (Fujii et al., 2007; Fabbro et al., 2014; Kong et al., 2006; Wang, 1998; Lin et al., 2006) are widely used. Researchers have further improved on these approaches, including co-culture in a hydroponic system, in sand as well as other substrates and nevertheless, have also utilized co-culture in field experiments, which could better simulate the natural state (Ahmad et al., 2013a,b; Xiao et al., 2012, 2013). Biological screening is very important in allelopathy research, and the results obtained are convenient, reliable and controllable (Cheng and Xu, 2013; Shi et al., 2014; Sun and Wang, 2015). However, biological screening has limitations, as it only can detect the allelopathic potential of a donor plant on a receptor plant but cannot reveal the exact mechanism of allelopathy. Therefore, additional experiments need to be conducted to determine the mechanism of allelopathy (Ding and Cheng, 2014).

Garlic, which is widely cultivated and consumed in China, has several desirable properties including insecticidal, antiviral, anticancer and antimicrobial effects that have attracted the wide attention of scholars and consumers (Iciek et al., 2009; Su and Cheng, 2009). In recent years, garlic has been proven to be a good preceding crop, thus indicating the potential allelopathic effects of this plant. Khan (Khan and Cheng, 2010; Khan et al., 2011) found that garlic root exudates played an important role in inhibiting the growth of pepper blight mycelia. Moreover, intercropping garlic with various crops can significantly improve both the microorganism populations and biochemical properties of the culture medium, together reducing the obstacles for continuous vegetable production (Liu et al., 2014; Wang et al., 2014; Xiao et al., 2013). Although allelochemicals in garlic have a significant influence on many species, their modes of action remain unclear. Recently, it was proposed that the effect of garlic on acceptor plant protective systems could be important to the allelopathy phenomenon (Ahmad et al., 2013a,b; Wang et al., 2015).

Previous studies on garlic's allelopathic effects on other plants have largely utilized field or pot conditions (Liu et al., 2014; Wang et al., 2014, 2015; Xiao et al., 2012, 2013), however, no research to date has suggested how garlic affects donor plants under hydroponic conditions. In addition, the allelochemicals of different vegetables are diverse, and they interact with target plants through different pathways, patterns and at different concentrations (Gniazdowska and Bogatek, 2005). Among these factors, the effect of concentration is key for the application of allelopathy to agricultural production. Similarly, the allelopathy of garlic root exudates depend on many factors, including the type and variety of receptor plant, the concentration of allelochemicals and the environmental conditions (Xiao et al., 2013; Zhou and Cheng, 2012; Zhou et al., 2007a,b). Many factors are uncertain under field conditions, which can affect the experiment and thus the results. However, hydroponic culture can help exclude possible microbial or nutrient interferences when assessing the allelopathic potential of a plant. Therefore, in the present study, we evaluated the allelopathic potential and concentration effect of garlic root exudates on pepper physiology by adopting a hydroponic co-culture method.

## RESULTS

### The effect of different pepper/garlic ratios (P/G) on the growth of pepper plants

After 30 days in hydroponic co-culture at four P/G ratios, the plant height, shoot fresh weight and root fresh weight of the pepper plants showed similar responses (Table 1). When the P/G ratio was 1:2, the fresh weight of roots and shoots increased by 21.9% and 29.3%, respectively, compared with the control (P/G ratio 1:0), with no significant impact on the pepper plant height. An increase in garlic to a P/G ratio of 1:4 significantly inhibited the height and weight of the aboveground of the pepper plants compared with the control (by 13.9% and 4.3%, respectively). However, the root fresh weight was slightly increased at the 1:4 P/G ratio but was significantly inhibited at the P/G ratio 1:6, by 33.3%, compared with the control. The root shoot ratio increased proportionately with the number of garlic plants.

**Table 1.**
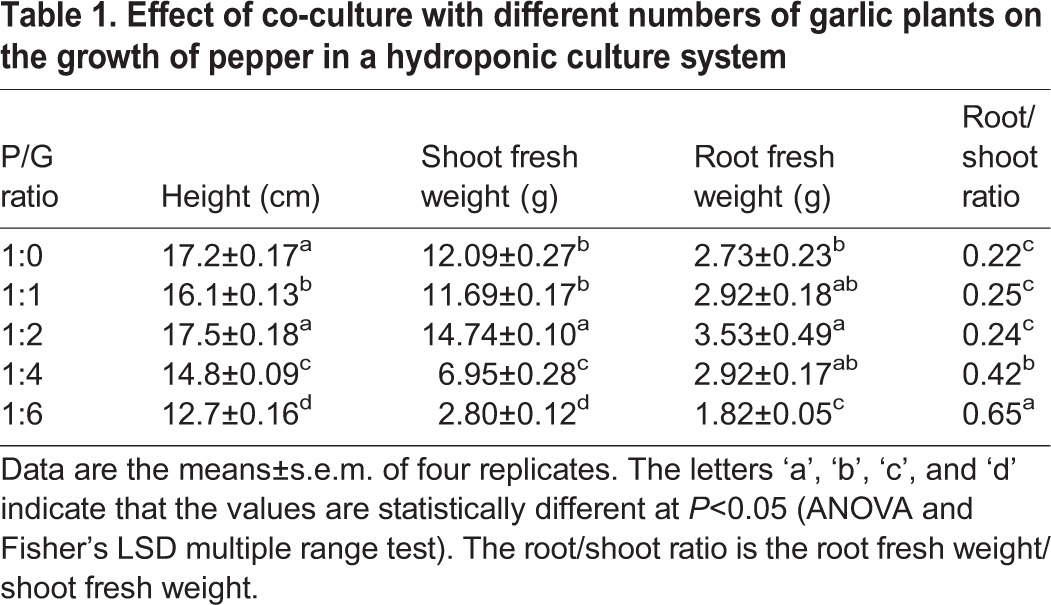
**Effect of co-culture with different numbers of garlic plants on the growth of pepper in a hydroponic culture system**

### The effect of different P/G ratios on the chlorophyll content of pepper

**Fig. 1.**
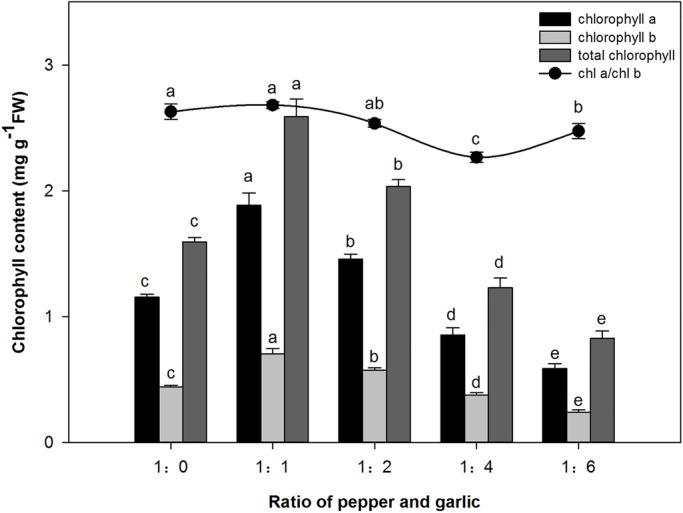
**Effect of co-culture with different numbers of garlic plants on the chlorophyll content of pepper in a hydroponic culture system.** Data are the means±s.e.m. of four replicates. The letters ‘a’, ‘b’, ‘c’, and ‘d’ indicate that the values are statistically different at *P*<0.05 (ANOVA and Fisher's LSD multiple range test).

After 30 days of co-culture, the chlorophyll a (chl-a), chlorophyll b (chl-b) and total chlorophyll contents of the pepper plants exhibited a dual response, increasing at a low P/G ratio (1:1 and 1:2) and decreasing at a P/G ratio of 1:4 and 1:6 (Fig. 1). When the P/G ratio was 1:1 or 1:2, the total chlorophyll content was significantly increased by 62.5% and 27.6%, respectively. However, at the P/G ratio 1:4, the total chlorophyll content was reduced by 22.8% compared with the control, decreasing significantly by 61.1% at the P/G ratio 1:6. The contents of chlorophyll a and chlorophyll b exhibited the same trend as that of total chlorophyll. In addition, the chl-a/chl-b value decreased with increasing P/G ratio and reached the lowest value at the P/G ratio 1:4 (Fig. 1).

### The effect of different P/G ratios on protective enzyme activities of pepper

**Fig. 2.**
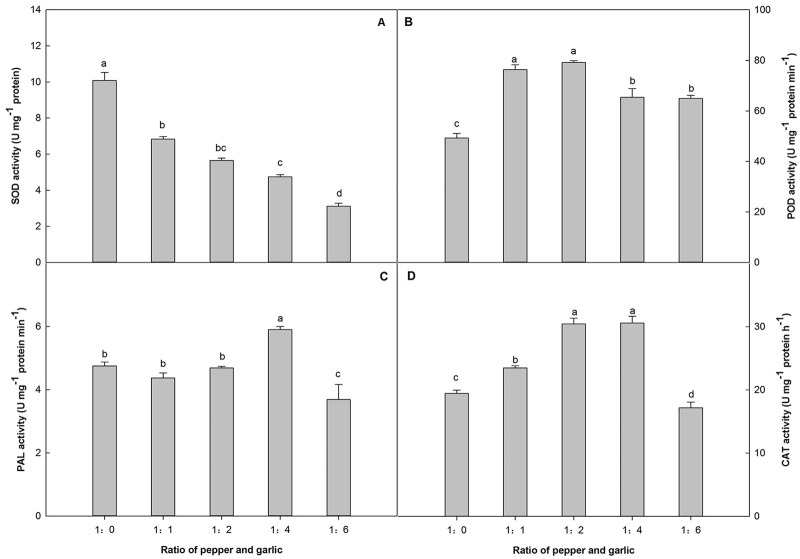
**Effect of co-culture with different numbers of garlic plants on the protective enzyme activities of pepper in a hydroponic culture system.** The effect of different P/G ratios on the SOD (A), POD (B), PAL (C) and CAT (D) activities of pepper. Data are the means±s.e.m. of four replicates. The letters ‘a’, ‘b’, ‘c’, and ‘d’ indicate that the values are statistically different at *P*<0.05 (ANOVA and Fisher's LSD multiple range test).

SOD activity in pepper leaves exhibited a decreasing trend with the increasing P/G ratio (Fig. 2A). Compared with the control, SOD activities in pepper plants decreased by 32.19%, 44.0%, 52.19% and 69.18% with increasing P/G ratio. The lowest value was observed at a P/G ratio 1:6.

The effect of the different P/G ratios on pepper peroxidase (POD) activity is presented in Fig. 2B. Compared with the control, an increasing P/G ratio resulted in enhanced activity: 54.80%, 60.57%, 32.69% and 31.73%. The highest level of POD activity was recorded at a P/G ratio 1:2.

PAL activity increased to a maximum value when the P/G ratio was 1:4, significantly higher than the control (24.14%). However, at the P/G ratio of 1:6, the garlic root exudates significantly inhibited PAL activity to a level below 77.5% of the control (Fig. 2C).

The effect of different P/G ratios on the catalase (CAT) activity of pepper plants is shown in Fig. 2D. CAT activity also showed an increasing trend, and a maximum level was achieved at the P/G ratios of 1:2 and 1:4, with increases of 56.7% and 57.3%, respectively. However, when the P/G ratio reached 1:6, the CAT activity dropped markedly to a level lower than that of the control.

### The effect of different P/G ratios on the MDA content of pepper

**Fig. 3.**
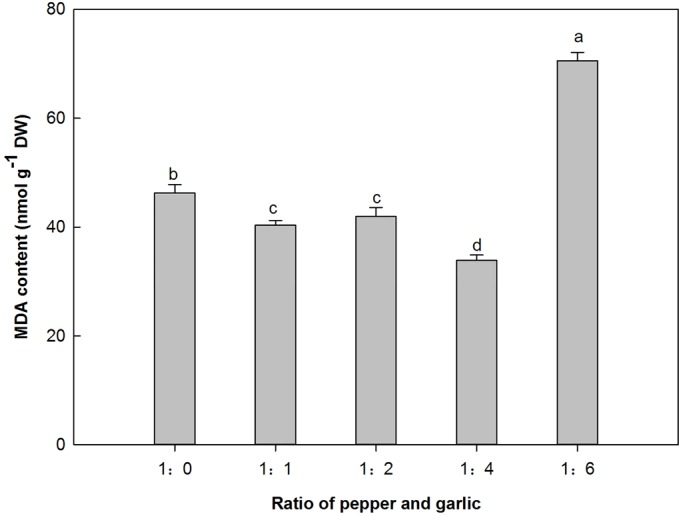
**Effect of co-culture with different numbers of garlic plants on the MDA content of pepper in a hydroponic culture system.** Data are the means±s.e.m. of four replicates. The letters ‘a’, ‘b’, ‘c’, and ‘d’ indicate that the values are statistically different at *P*<0.05 (ANOVA and Fisher's LSD multiple range test).

The MDA content of pepper was affected by an increasing P/G ratio, and a significantly low level of MDA content was observed at the P/G ratio of 1:4, which was reduced by 26.8% compared with the control. However, as the P/G ratio increased to the maximum (1:6), a significantly high MDA level was recorded, 52.49% higher than the control (Fig. 3).

## DISCUSSION

Seed germination and seedling growth studies under laboratory conditions are widely used to evaluate the action of allelochemicals on crops in biological assays and can indicate the initial visible symptoms of allelochemical-induced stress (Bauer et al., 2012; Kato-Noguchi et al., 2012; Pellissier, 2013). In the present study, controlled environmental conditions, including pH, air and nutrients in solution, were employed to avoid possible interferences of soil origin. Therefore, this co-culture system is considered to be a better approach toward understanding the possible effects of garlic root exudates on the growth and physiology of pepper plants.

When environmental conditions trigger a signal, plants respond via phenotypic and physiological changes that can be correlated to the signal if they are carefully observed and understood. In this study, we observed the effects of different P/G ratios, which could be interpreted as different concentrations of garlic root exudates, on pepper plants, and the results revealed a dose-dependent effect. In response to low concentrations of garlic root exudates (P/G ratios of 1:1 and 1:2), the pepper shoot fresh weight, root fresh weight and plant height increased, peaking at the 1:2 ratio (Table 1). One reason for this finding could be that the garlic root exudates promoted the root growth of the pepper plants and thus the absorption of mineral elements, allowing for enhanced assimilation of nutrients by the leaves and resulting in the increased fresh weight of the aboveground parts (Xiao et al., 2012). Moreover, garlic root exudates may regulate endogenous hormones, and these hormones may have influenced the growth of the pepper plants (Oracz et al., 2012).

The chlorophyll content of the pepper plants was highest when the P/G ratio was 1:1 (Fig. 1), which indicated that low concentrations of garlic root exudates could increase the chlorophyll content of pepper. This may be due to the allelochemicals in the garlic root exudates participating in the chlorophyll biosynthetic pathway, thus enhancing its content in the pepper plants (Shi et al., 2009). Increases in chlorophyll content would promote photosynthesis, resulting in more photosynthate, energy and dry matter accumulation in the pepper plants. The results indicate that a low concentration of garlic root exudates promoted the growth of pepper seedlings, and this result is further supported by the findings of sufficient energy supply for the accumulation of dry matter. These results are consistent with the findings of Zhou (2007b).

However, we also observed a significant reduction in pepper plant height, shoot and root fresh weight and chlorophyll content when the P/G ratio was 1:4 (Table 1, Fig. 1), indicating that a high concentration of allelochemicals inhibits photosynthesis and plant growth by reducing the chlorophyll content. These results are in agreement with the findings of Zeng et al. (2009), who observed that shoot extracts of *Artemisia judaica* caused a dose-dependent reduction in the chlorophyll content of lettuce leaves under their assay conditions. Thus, the high concentrations of garlic root exudates may have stressed the pepper plant. In addition, the root/shoot ratio (Table 1) increased proportionately with the number of garlic bulbs and reached highest when the P/G ratio was 1:6, indicating that the growth of the aboveground part is more strongly affected by a high concentration of garlic root exudates than the growth of belowground parts. Previous studies have also reported that the root/shoot ratio increases under stress conditions, with greater negative effects on above- versus belowground parts. This is because it is difficult for shoots to grow normally without sufficient water and nutrient supplies, whereas roots will accelerate growth to absorb more water and nutrients for survival, resulting in an increase in the root/shoot ratio under stress conditions (Wallihan and Garber, 1968; Edgren and Iyer, 1979; Turner, 1992). However, further study is needed for an in-depth understanding of this process.

In the current study, the allelochemicals in the garlic root exudates not only functioned as signals but also as indirect ROS scavengers for the pepper plants. At a low concentration (P/G ratio of 1:1), the MDA content decreased compared with the control and reached the lowest level at a P/G ratio of 1:4, indicating that garlic root exudates could decrease the extent of membrane lipid peroxidation of pepper plants. This finding indicates that the appropriate concentration of garlic root exudates can induce the activity of the associated protective enzymes (Fig. 2). Moreover, the phenolic compounds in garlic root exudates may help pepper plants to eliminate ROS in the leaves. As a result, the damage due to membrane lipid peroxidation is reduced. With regard to the possible roles of oxidative stress caused by garlic root exudates on pepper plants, changes in the main antioxidant enzymes were also investigated. The activity of associated protective enzymes (POD, CAT and PAL) fluctuated at different inflection points but revealed overall similar patterns, increasing first and decreasing thereafter, with the exception of SOD. These results may be because the effect of the garlic root exudates is indirect and occurs via ROS level regulation in cells; thus, the related genes may be regulated by antioxidant response elements (Oracz et al., 2007). In contrast, MDA, which is an important stress indicator in plants, showed the opposite trend, decreasing first and then increasing, which reflects the synergy of the protective enzyme system in pepper plants (Wang et al., 2015). Under low concentrations of garlic root exudates, the allelochemicals in the exudates maximally induced the activities of POD, CAT, and PAL. However, the growth of the pepper plants was significantly inhibited by the maximum concentration of allelochemicals at the P/G ratio of 1:6, reducing the activities of SOD, CAT and POD to their lowest levels. The toxic effect of the garlic root exudate allelochemicals on the activity of protective enzymes might be caused by disrupting their structures (Polak et al., 2012). At the highest reduction in protective enzyme activity at the P/G ratio of 1:6, irreversible cell membrane damage occurred, leading to a burst of ROS and membrane peroxidation. Under this condition, the MDA content was significantly increased, which demonstrated that serious cell membrane lipid peroxidation occurred in the pepper plants. Under these circumstances, the pepper biomass was significantly reduced to its minimum value, demonstrating serious inhibition of plant growth. A possible reason may be due to the presence of phenolic compounds, such as 2, 6-diisopropyl phenol, 2, 6-di-tert-butyl-4methylphenol and 2, 2′- methylene double (4-methyl-6-tertiary butyl) phenol, in the garlic root exudates (Liu et al., 2011; Zhou and Cheng, 2012), which disperse in all types of tissues and organs in the plant and also serve as signals for regulating plant growth and as scavengers for eliminating ROS (Smirnoff, 2005).

Additionally, the potential energy costs to the pepper plants and the mechanisms of pepper plant adaptation to the allelopathy of garlic root exudates cannot be ignored. Under optimal circumstances, most of the energy acquired by photosynthesis is used for basic requirements, with only a small proportion (10–40%) used directly for biomass accumulation (Amthor, 2000; Jacoby et al., 2011). However, the energy allocated to growth is reduced or redistributed toward defence when stress occurs (Munns and Gilliham, 2015). This switch can explain the observed growth inhibition in pepper exposed to a high concentration of garlic root exudates, which would lead to a redistribution of the pepper plant's energy from growth to stress defence (Table 1). Moreover, pepper plants may excrete more autotoxins, such as N-phenyl-2-naphthylamine and phthalic acid (Sun and Wang, 2015), when stimulated by a high concentration of garlic root exudates, which may enhance the deleterious effect on the pepper plant itself (Zhang et al., 2010). Moreover, this behaviour has potential energy costs, resulting in growth inhibition.

Competition for resources is also an important aspect that cannot be ignored. In our experiment, different quantities of garlic may have contributed not only to increasing concentrations of garlic root exudates but also to differential growth conditions in the co-culture system, including nutrition and space. Although the experimental design accounted for these factors, it is certain that each pepper plant suffered competition for more space and nutrition with an increasing number of garlic plants. We cannot exclude that such energy costs to the pepper plants (Munns and Gilliham, 2015) resulted in growth inhibition, which is a disadvantage of this co-cultured method that could not be avoided in this experiment.

These physiological alterations in pepper plants reveal how and where garlic interacts with a target plant through allelopathy. Low concentrations of garlic allelochemicals tend to be beneficial for increasing pepper growth and thus would alleviate some of the possible cropping obstacles in pepper production. However, at a certain concentration, these exudates also hinder pepper growth by altering its physiology, which indicates that there might be hazards when intercropping garlic with pepper plants if the garlic accounts for a high proportion in the cropping system. However, the potential allelopathic effects of garlic on pepper in a managed culture technique (hydroponic co-culture system) may not be representative of field cultivation. In a hydroponic culture system, allelopathy is clearly evident at an actual concentration of allelochemicals; however, this concentration may not exert inhibition under field conditions. Therefore, with regard to the possible effects of garlic on pepper, a field experiment over three years was also conducted to investigate the effects of intercropping with different numbers of green garlic plants on pepper, and a dose-dependent effect was also observed (our unpublished results). In the future, these results can be translated to real soil conditions by quantifying the concentration of allelochemicals in garlic root exudates in the medium. Molecular approaches may help to identify the proteins and genes involved in these physiological responses to the surrounding environment. Appropriate intercropping ratios that provide low concentrations of garlic allelochemicals can be utilized to alleviate the cropping obstacles that hinder pepper cultivation; however, to achieve sustainably improved plant growth, a relatively high proportion of garlic should be avoided.

## MATERIALS AND METHODS

### Plant material and growth conditions

Garlic cv. Gailiang (G064) was obtained from the Department of Horticulture, Northwest A&F University, Yangling, Shaanxi, China. Twenty fresh and uniformly sized bulbs (approximately 5 cm diameter, 4 cm height and 45 g weight) were sown in sterilized perlite (autoclaved at 125 Pa/121°C for 30 min) in a hard plastic culture pot (40 cm×30 cm×12 cm) and incubated at 20°C to yield a rooting culture. The medium was kept moist with distilled water. When new roots emerged (approximately 5 cm long) one week later, the garlic bulbs were uprooted, rinsed with tap water to remove the perlite, sterilized with 0.05% potassium permanganate for 20 min, rinsed with distilled water and grown hydroponically in Hoagland's nutrient solution together with pepper seedlings for 30 days.

The seeds of pepper cv. Nongcheng No. 2 were sterilized in hot water (55°C) and stirred for fifteen minutes and then rinsed three times with distilled water. The plant growth medium, which was commercially procured, contained peat moss for optimum growth and was autoclaved (125 Pa/121°C) for 30 min. The medium was placed in plastic pots (8 cm×8 cm×10 cm), and the pepper seeds were planted, followed by shower irrigation and transfer to a growth chamber at 25°C/20°C day/night temperatures, 70% humidity level and a 12-h light period. On alternate days, shower irrigation was performed to ensure uniform seedling growth. At the four-leaf growth stage, uniformly sized seedlings were selected for further experiments and transplanted to the hydroponic culture medium. The selected pepper seedlings were rinsed with tap water to remove the perlite, sterilized with 0.05% potassium permanganate for 20 min, rinsed with distilled water and grown hydroponically in Hoagland's nutrient solution with garlic plants for 30 days.

### Experimental design

The experiment was conducted in April 2013 at the Horticultural Experiment Station (N 34°16′, E 108°4′), College of Horticulture, Northwest A&F University, Yangling, Shaanxi Province, China. Each hydroponic system was mainly composed of a plastic pot (40 cm×30 cm×12 cm) and an air pump (220 V, 80 W) with a time switch; the pump was used to periodically recycle the air (20 min/h). Five treatments were included: two pepper plants were co-cultured with 0, 2, 4, 8 and 12 garlic bulbs per pot to give pepper/garlic ratios (hereafter referred to as the P/G ratio) of 1:0 (control), 1:1, 1:2, 1:4 and 1:6, respectively. Each treatment was replicated four times. Each pot contained 6 litre Hoagland's nutrient solution (Zhang, 2003), which was refreshed every 10 days. The pH was adjusted to 6.0 using H_2_SO_4_ or NaOH.

### Plant growth analysis

After 30 days of co-culture, morphological and physiological indices were evaluated using two pepper plants from each replication. The plant height and the fresh weight of roots and shoots were measured with a ruler and a balance, respectively, and the corresponding data were recorded.

### Determination of chlorophyll contents

Chlorophyll extraction from each sample was performed according to the method of Hemavathi (Hemavathi et al., 2010). Acetone (80% v/v) was used as the solvent. Briefly, 0.2 g fresh weight of fragmented leaf tissue was placed in a glass tube containing 10 ml 80% acetone. The extracted liquid was placed in darkness for 24 h, and the volume was brought to a total of 20 ml with 80% acetone. Chlorophyll determination was performed with a spectrophotometer (UNIC 2100) at 645 and 663 nm. The chlorophyll a and b and total chlorophyll contents were calculated following the equations of Gao (2006).

### Antioxidative enzyme assay

To evaluate antioxidative enzymes, fresh leaf tissue (0.5 g) was homogenized in an ice bath in 10 ml of chilled 200 mM phosphate buffer (pH 7.8) containing 1% (w/v) soluble polyvinyl pyrrolidone (PVP). The homogenate was centrifuged at 10,000 ***g*** for 20 min at 4°C, and the supernatant was used to assay the enzymatic activity.

The soluble protein in the supernatant was estimated as described by Shi et al. (2014) using bovine serum albumin (BSA) as a standard. The sample (20 μl) was mixed with 80 μl phosphate buffer (pH 7.8) and 2.9 ml Coomassie Brilliant blue and vortexed, and the absorbance at 595 nm was recorded after 2 min.

The activity of superoxide dismutase (SOD) was determined according to Ali et al. (2014). The reaction mixture contained 100 µl of enzyme extract, 100 µl of 1.31 M riboflavin and 3 ml of SOD buffer. The reaction was performed at 25°C under fluorescent lamps (40 W) for 15 min until the solution turned dark, indicating that formazan had formed as a result of nitroblue tetrazolium (NBT) photoreduction. The same reaction mixture kept in the dark without illumination was used as a control, and a mixture lacking the leaf extract was used as a blank. Absorbance was recorded at 560 nm using a UV-visible spectrophotometer (UNIC 2100). One unit of SOD was defined as the amount of enzyme inhibiting NBT photoreduction by 50% in comparison with the sample lacking the plant extract. The SOD activity is presented as U mg^−1^ protein.

The activities of peroxidase (POD), catalase (CAT) and phenylalanine ammonia-lyase (PAL) were determined following the method of Wang et al. (2015). The POD reaction mixture consisted of 0.1 ml enzyme extract, 2.9 ml phosphate buffer (pH 7.0), 0.2% methyl catechol and 0.3% H_2_O_2_. The change in absorbance was recorded at 485 nm after 3 min with a spectrophotometer. The POD activity is presented as OD 485 nm min^−1^ mg^−1^ protein.

The CAT reaction mixture contained 0.1 ml enzyme extract, 1.9 ml of 200 mM phosphate buffer (pH 7.0), and 1 ml 0.3% H_2_O_2_, and enzyme activity was assayed by measuring the reduction of H_2_O_2_ at 240 nm for 3 min with a spectrophotometer. The activity of CAT is presented as OD 240 nm h^−1^ mg^−1^ protein.

The PAL reaction mixture contained 0.3 ml enzyme extract, 2.7 ml of borate buffer solution (pH 8.8), and 1 ml 0.02 M phenylalanine, and the mixture was incubated in a water bath at 37°C for 1 h. Absorbance was measured at 290 nm using a UV-visible spectrophotometer.

### Determination of the malondialdehyde content

The malondialdehyde (MDA) content was determined by the thiobarbituric acid reaction (Shi et al., 2014), with slight modifications. An extract was prepared using the same method as for determining antioxidant enzyme activity. The reaction mixture contained 1.5 ml extract and 2.5 ml 0.5% thiobarbituric acid (TBA, w/v). The mixture was incubated at 100°C for 20 min before being cooled in an ice bath. After centrifugation at 7888 ***g*** for 10 min, the absorbance of the supernatant was measured at 450 nm, 532 nm and 600 nm using a spectrophotometer. The MDA content was calculated using the formula: MDA content (μmol/g^−1^ DW)=[6.452×(OD_532_-OD_600_)-0.559×OD_450_]×8/(DW×1.5). (DW, dry weight).

### Statistical analysis

The experiment was performed according to a completely randomized design (CRD).

The values of all the data are represented as the mean±s.e.m. of four independent replicates.

Analyses of variance (ANOVA) was performed to determine significant differences according to Fisher's least significant difference (LSD) multiple range test at *P*<0.05. STATISTICA 8.0 software (Informer Technologies) was used.
